# Anti-Biofilm and Antibacterial Activities of *Cycas media* R. Br Secondary Metabolites: In Silico, In Vitro, and In Vivo Approaches

**DOI:** 10.3390/antibiotics11080993

**Published:** 2022-07-24

**Authors:** Nashwah G. M. Attallah, Omnia Momtaz Al-Fakhrany, Engy Elekhnawy, Ismail A. Hussein, Moataz A. Shaldam, Najla Altwaijry, Moneerah J. Alqahtani, Walaa A. Negm

**Affiliations:** 1 Department of Pharmaceutical Science, College of Pharmacy, Princess Nourah Bint Abdulrahman University, P.O. Box 84428, Riyadh 11671, Saudi Arabia; ngmohamed@pnu.edu.sa (N.G.M.A.); naaltwaijry@pnu.edu.sa (N.A.); 2 Department of Pharmaceutical Microbiology, Faculty of Pharmacy, Tanta University, Tanta 31527, Egypt; omina.elfakharany@pharm.tanta.edu.eg; 3 Department of Pharmacognosy and Medicinal Plants, Faculty of Pharmacy (Boys), Al-Azhar University, Cairo 11884, Egypt; 4 Department of Pharmaceutical Chemistry, Faculty of Pharmacy, Kafrelsheikh University, Kafr El-Sheikh 33516, Egypt; dr_moutaz_986@pharm.kfs.edu.eg; 5 Department of Pharmacognosy, College of Pharmacy, King Saud University, P.O. Box 2457, Riyadh 11451, Saudi Arabia; mjalqahtani@ksu.edu.sa; 6 Department of Pharmacognosy, Faculty of Pharmacy, Tanta University, Tanta 31527, Egypt

**Keywords:** ginkgetin, phytotherapy, sotetsuflavone, interleukins, tumor necrosis factor-alpha, virulence

## Abstract

*Enterococcus* species possess many virulence factors that have an essential role in exacerbating the infections caused by them. The current study aimed to evaluate the effect of the secondary metabolites ginkgetin (GINK) and sotetsuflavone (SOTE), isolated from *Cycas media* R. Br dichloromethane fraction, on *Enterococcus faecalis* (*E. faecalis*) isolates for the first time. The antibacterial and antivirulence activities of the isolated compounds were investigated using docking studies and in vitro by determination of the minimum inhibitory concentrations (MICs). Additionally, flow cytometry and scanning electron microscope (SEM) were utilized to assess the effect of SOTE on the tested bacteria. Moreover, crystal violet assay and qRT-PCR were used to test the effect of SOTE on the biofilm-forming ability of *E. faecalis* isolates. In addition, a systemic infection model was utilized in vivo to investigate the antibacterial activity of SOTE. We found that both GINK and SOTE showed a good affinity for the five proteins enrolled in the virulence of *E. faecalis*, with SOTE being the highest, suggesting the possible mechanisms for the antivirulence activity of both ligands. In addition, SOTE exhibited a higher antibacterial activity than GINK, as the values of the MICs of SOTE were lower than those of GINK. Thus, we performed the in vitro and in vivo assays on SOTE. However, they did not exhibit any significant variations (*p* > 0.05) in the membrane depolarization of *E. faecalis* isolates. Moreover, as evaluated by SEM, SOTE caused distortion and deformation in the treated cells. Regarding its impact on the biofilm formation, it inhibited the biofilm-forming ability of the tested isolates, as determined by crystal violet assay and qRT-PCR. The in vivo experiment revealed that SOTE resulted in a reduction of the inflammation of the liver and spleen with an increase in the survival rate. SOTE also improved the liver-function tests and decreased tumor necrosis factor-alpha using immunostaining and the inflammation markers, interleukins (IL-1β and IL-6), using ELISA. Thus, we can conclude that SOTE could be a promising compound that should be investigated in future preclinical and clinical studies.

## 1. Introduction

*Enterococci* are Gram-positive cocci commonly present in the gastrointestinal tracts of humans. Although they are considered a prominent member of the human microbiome, they can produce many infectious diseases [1]. *Enterococcus faecalis* can cause various infections, including endocarditis, urinary tract infections, prostatitis, intra-abdominal infection, cellulitis, and wound infection, as well as concurrent bacteremia. Moreover, they are an important cause of nosocomial infections. *Enterococci*, especially *E. faecalis,* a widespread species in hospitals and clinical settings, are now the third most common nosocomial pathogen [2]. 

Unfortunately, *E. faecalis* is resistant to many drugs of commonly utilized antibiotics. These antibiotics like vancomycin, beta-lactams, gentamicin, and erythromycin [3]. In addition to the antibiotic resistance problem, *E. feacalis* possesses various virulence factors that enable it to colonize human tissues. Biofilm formation is a significant virulence factor of *E. feacalis.* Biofilm is a population of bacterial cells that grow on living or nonliving surfaces and are integrated into a self-produced matrix of several macromolecules such as proteins, polysaccharides, lipids, and DNA [4]. 

Biofilms protect bacteria that are embedded in them from environmental stress, detergents, and antimicrobial agents. Thus, biofilm makes eradicating bacteria extremely difficult, owing to the high level of antibiotic resistance [5]. In contrast to the planktonic bacterial cells, cells embedded in biofilm exhibit multidrug resistance and could be untreatable with conventional antibiotics. Regarding *E. feacalis*, it can cause various biofilm-related infections, such as catheter-related infections [2]. 

Consequently, there is a high and urgent need to recognize novel treatment strategies for infections caused by *E. feacalis*. Phytotherapy is an alternative medicine approach using plant extracts or the compounds isolated from them for treatment. It is a non-antibiotic strategy that has a long history, spanning centuries, of being used to prevent or treat different infectious diseases [6]. In addition, medicinal plants have many bioactive substances that can protect the plants themselves from various pathogens and, thus, could have the ability to prevent and/or treat human infections [6].

The plant genus *Cycas* is part of Cycadophyta, a very old lineage that is not closely linked to palms, ferns, trees, or any other modern group of plants. They were distributed almost all over the world. The only recognized extant genus in the family Cycadaceae is *Cycas*, which is also its genus. There are about 115 recognized and accepted species in the genus *Cycas* [7]. The first cycad species to be described was *Cycas circinalis*, an Indian species that serves as the type for the generic name *Cycas*. *Cycas revoluta* is the species of cycads that is best-known and is also widely cultivated [8]. *Cycas media* R. Br is a cycad that can reach heights of up to three meters and, on rare occasions, six meters. It is a plant with a palm-like look that typically produces unbranched stems with rosettes of leaves, each 70 to 180 cm long. *C. media* is native to the tropics of eastern Queensland, frequently seen close to the coast. *C. media* is a plant with cones that resembles *Cycas circinalis* in shape. *C. media* is a hardy plant frequently grown in rockeries or gardens. It is appropriate for tropical areas with a year-round dry climate. It grows easily and can withstand dry periods [7,8,9].

*Cycas* is a dioecious plant that possesses a diverse range of bioactive compounds corresponding to many chemical classes. These substances like flavonoids, terpenoids, norisoprenoids, lignans, biflavonoids, aromatic acids, sterols, and others [8,10]. Among the different species of *Cycas*, *Cycas media* R. Br have few phytochemical and biological investigations in the literature, an issue which inspired us to conduct this study.

Herein, we isolated the major compounds ginkgetin (GINK) and sotetsuflavone (SOTE) from *C. media* R. Br highly yielded dichloromethane fraction for the first time. Moreover, we investigated the antivirulence activity of GINK and SOTE on *E. faecalis* by docking studies. In addition, we intended to perform in vitro and in vivo antibacterial and antivirulence studies for the more active compound on *E. faecalis* clinical isolates.

## 2. Materials and Methods

### 2.1. General 

The utilized media, such as Mueller–Hinton agar (MHA) and Mueller–Hinton broth (MHB), as well as chemicals, were purchased from Oxoid, Hampshire, UK, and Merck, Kenilworth, New Jersey, USA. For column chromatography (CC), we employed Silica gel F254 (Merck, 70–230 mesh) and Sephadex LH-20 (Sigma Aldrich Chemical Co., St. Louis, MO, USA). 

NMR spectra for ^1^H and ^13^C were acquired using a JEOL ECA500-II-NMR spectrometer. The NMR sample was dis-solved in DMSO-*d_6_.* Solvent resonances were used to normalize the chemical changes. The ESI-MS was performed using the USA Mass Spectrometer, Xcalibur 2.1 software, and ISQ Quantum Access MAX Triple Quadrupole system from Thermo Scientific, MA, USA.

### 2.2. Plant, Extraction, and Isolation

*Cycas media* R. Br leaves were selected from Al-Orman Botanical Garden on 15 July 2021. The plant was identified by Dr. Esraa Ammar, Plant Ecology, Tanta University. A voucher sample (PG-G-107-E) was deposited at the Herbarium of the Faculty of Science, Tanta University. The leaves were dried at room temperature for 12 days, then in an oven for two days at 40 °C and powdered. The powder (370 g) was extracted by methanol (CH_3_OH) (5 L × three times); the extract was concentrated using a rotary evaporator to yield 40.12 g of *C. media*. The total *C. media* extract (40 g) was resuspended in CH_3_OH: H_2_O (1:1 *v*/*v*), then partitioned with different solvents Pet-ether, dichloromethane, ethyl acetate, and finally *n*-butanol saturated with water to yield 10.13, 12.88, 2.02, and 7.18 g residues, respectively (Scheme S1).

The highly yielded dichloromethane (DCM) fraction (7 g) was then chromatographed (ϕ 4 × 80 cm, 210 g silica, collected fraction 50 mL) using gradient elution, starting with 100% *v*/*v n*-hexane, and increasing polarity with DCM, until 100% DCM was reached, and then the polarity started to increase with CH_3_OH until four significant fractions were obtained (D1: D4): Fr. D1 (*n-*hexane–DCM 8:92% *v*/*v* eluate), Fr. D2 (DCM–CH_3_OH; 92:8% *v*/*v* eluate), Fr. D3 (DCM–CH_3_OH; 88:12% *v*/*v* eluate), and Fr. D4 (DCM–CH_3_OH; 86:14% *v*/*v* eluate). Fr. D2 (0.96 g) was chromatographed using silica gel, and subfractions (eluted with CHCl_3_–CH_3_OH; 94:6% *v*/*v*) were collected and then purified using Sephadex LH-20 eluted with 100% methanol to obtain a yellow amorphous powder referred to as compound I. Fr. D3 (1.89 g) was subjected to isocratic CC (CHCl_3_–CH_3_OH; 90:10) and then purified using Sephadex to yield a yellow powder, referred to as compound II (Scheme S2).

### 2.3. Bacterial Isolates

Twenty-three *E. faecalis* clinical isolates were obtained from different specimens from the laboratories of Tanta University Hospitals. Different biochemical tests, such as Gram staining, catalase test, as well as esculin hydrolysis on bile-esculin agar, were used for bacterial identification [11]. The identification was confirmed using matrix-assisted laser desorption ionization-time of flight mass spectrometry (MALDI-TOF MS). *Enterococcus faecalis* strain (ATCC 19433) was used as a standard isolate.

### 2.4. Animals

Forty-five male albino rats with weights ranging from 120 to 150 g and at the age of eight weeks old were acquired from the Faculty of Veterinary Medicine, Cairo University, Egypt. The animals were left for seven days to adapt to the environmental conditions. Moreover, they were retained on a regular feed, *ad libitum*, and given water. Our in vivo experiment followed the standards of using laboratory animals of the Faculty of Pharmacy Research Ethical Committee, Tanta University (code number: TP/RE/04-22-P-011).

### 2.5. Molecular Docking

*E. faecalis* cytolysin regulator CylR2 (Code:1UTX) [12], *E. faecalis* collagen-binding subdomain ACE19 (Code: 2OKM) [13], adhesin domain of PrgB from *E. faecalis* (Code: 6GED) [14], and Enterococcal surface protein ESP (code: 6ORI) [15] were obtained from the protein data bank (PDB), while the model (A0A7H0FPW4) for *E. faecalis* gelatinase enzyme EFGE was retrieved from the Swiss-model portal [16]. The docking study was carried out on SOTE and GINK using AutoDock Vina [17]. Ligand structures were drawn into Marvin Sketch V22.2 [18], and the most energetically favored conformer was exported as a (*.pdb) file format. The procedure for docking simulation was performed according to our previous study steps [19]. The centers and sizes of the grid boxes used to define the active site for each receptor are shown in Table 1. The 3D visualization and 2D schematic presentation were generated by Pymol [20] and LigPlot^+^ V2.2.4 [21], respectively.

### 2.6. In Vitro Antibacterial Activity

#### 2.6.1. Antibiotic Susceptibility Profile of the Tested Isolates

The Kirby–Bauer method was utilized to assess the antibiotic susceptibility of the tested isolates as previously described [22]. The utilized antibiotic discs (Oxoid, UK) were gentamicin (CN, 120 µg), erythromycin (E, 15 μg), ampicillin (AMP, 10 μg), amoxicillin/clavulanic acid (AMC, 20/10 μg), chloramphenicol (C, 30 µg), ciprofloxacin (CIP, 5 µg), tetracycline (TE, 30 µg), vancomycin (VA, 30 µg), and linezolid (LZD, 30 µg). In brief, plates containing MHA were inoculated with bacterial suspensions by sterile swabs. Then, antibiotic discs were located on the surface of the MHA plates and incubated overnight at 37 °C. Finally, the produced inhibition zones were measured, and the susceptibility to the utilized antibiotics was interpreted using interpretation tables [22].

#### 2.6.2. Susceptibility Testing of GINK and SOTE

The agar well diffusion method [9,23] was utilized for preliminary screening of the antibacterial activity of GINK and SOTE against *E. faecalis* clinical isolates. One hundred microliters of the bacterial suspensions were distributed onto the surface of MHA plates. Then, four wells were made in the agar by a cork-borer, and each well was filled with 100 μL (1024 µg/mL) of each of the following: GINK and SOTE (solubilized in dimethyl sulfoxide or DMSO). The other two wells contained DMSO (10%) as a negative control and vancomycin (30 μg/mL) as a positive control [22]. The plates were then inspected for the appearance of inhibition zones around the wells.

We also determined the minimum inhibitory concentrations (MICs) of GINK and SOTE using the broth microdilution method, as previously described [24], in 96-well microtitration plates. The MIC values of GINK and SOTE (solubilized in DMSO concentration ≤ 1%) were identified for each bacterial isolate as the lowest extract concentration which exhibited complete inhibition of the bacterial growth (i.e., absence of turbidity). They were diluted using serial two-fold dilution in MHB and incubated overnight at 37 °C for 24 h.

#### 2.6.3. Impact of SOTE on the Membrane Depolarization

The membrane depolarization was studied before and after treatment with 0.5 MIC of SOTE by DiBAC4(3) [25,26]. After centrifugation of the bacterial suspensions, the pellets were resuspended in phosphate-buffered saline (PBS). Then, 5 μg/mL DiBAC4(3) was used. The staining of the bacterial cells was analyzed using the FACSVerse flow cytometer (BD Biosciences, Franklin Lakes, New Jersey, NJ, USA).

#### 2.6.4. Studying the Effect of SOTE on the Bacterial Morphology

The morphology of *E. faecalis* clinical isolates was studied before and after treatment with 0.5 MIC of SOTE using a scanning electron microscope (SEM) (Hitachi, Tokyo, Japan) [27]. The pellets obtained after centrifugation of the bacterial suspensions were first prefixed using glutaraldehyde (2.5%) in 0.05-M cacodylate buffer. Then, fixation with osmium tetroxide (1%) in Ryter-Kellenberger buffer was carried out. The samples were then placed on aluminum stabs and finally sputter-coated with gold to be examined by SEM.

#### 2.6.5. Anti-Biofilm Activity of SOTE

The biofilm-forming ability of the isolates (non-biofilm formers, weak, moderate, or strong) was determined using crystal violet assay in 96-well microtitration plates. The optical density (OD) at 490 nm was detected using an ELISA reader (Sunrise Tecan, McLean, Virginia, VA, USA). The isolates were grouped into four groups based on their determined OD values as previously reported [28]. Then, the impact of SOTE (at 0.5 MIC value) on the biofilm formation was evaluated. In addition, the viability of the adherent cells in the wells of the microtitration plates was assessed, before and after treatment, by counting the number of colony-forming units (CFU) per milliliter [29]. 

#### 2.6.6. Quantitative Real-Time Polymerase Chain Reaction (qRT-PCR)

qRT-PCR was utilized to determine the expression level of the virulence genes related to biofilm formation. These genes include enterococcal surface protein (*esp*), enterococcal cytolysin (*cyl*L), cytolysin operon (*cyl*A), aggregation substance (*agg*), cell wall adhesins (*efa*A), and enterococcal surface adhesion (*ace*). Total RNA was extracted from the *E. faecalis* isolates using the Purelink™ RNA Mini Kit (Thermo Scientific, Waltham, Massachusetts, MA, USA) as described by the manufacturer. Then, RNA was transformed into cDNA using a power™ cDNA synthesis kit (iNtRON Biotechnology, Korea) according to the manufacturer’s instructions. 

Finally, qRT-PCR was carried out using Rotor-Gene Q 5plex (Qiagen, Hilden, Germany). The primer sequence of the housekeeping gene (16S rRNA) and the sequences of the utilized primers in the present study are listed in Table S1 [30]. The 2^−ΔΔCt^ method was used in the current study to quantify the relative gene expression. The level of the gene expression of the isolates before treatment with SOTE was considered one. Fold changes were statistically significant when there were two or more fold changes (either increased or decreased) [31].

### 2.7. In Vivo Antibacterial Activity

#### 2.7.1. Experimental Model

Animals were randomly grouped into three groups, then all of them were administered bacterial suspension of *E. faecalis* (0.2 mL, 2.0 × 10^7^ CFU/mL) for two days by subcutaneous (SC) injection to induce systemic infection. The three groups were as follows [24,32]:

Group I (*E. faecalis* group): fifteen rats were administered sterile water intraperitoneal (IP) injection for 20 days.

Group II (gentamicin treated group): fifteen rats were administered gentamicin (2 mg/kg) via IP injection for 20 days.

Group III (SOTE treated group): fifteen rats were administered 10 mg/kg SOTE via IP injection for 20 days.

All animals received their first dose of treatments 24 h after the second SC bacterial injection. Five rats in each group were monitored for 20 days to assess the survival rate. After ten days, the remaining rats were anesthetized and euthanized by cervical dislocation, liver and spleen were collected for histological and immunohistochemical investigations, and blood samples were instantly collected. In addition, the bacterial burden in liver and spleen tissues was determined based on the count of CFU/g tissues.

#### 2.7.2. Liver Function Tests

Serum was utilized, in the current study, for assessment of liver function using commercial kits of ELISA. The levels of alanine aminotransferase (ALT) (ab234579, Abcam, Cambridge, UK), aspartate aminotransferase (AST) (ab263883, Abcam, Cambridge, UK), albumin (ab108789, Abcam, Cambridge, UK), total proteins (BioRad, Hercules, CA, USA), and bilirubin (MBS730053, MyBioSource, San Diego, CA, USA) were measured in all groups [24].

#### 2.7.3. Histological Assessment

Hematoxylin and eosin (H&E), in addition to Masson’s trichrome staining, were performed on the liver and spleen tissues and were preserved in 10% formalin of the different tested groups. Then, photos were obtained using a digital camera to investigate the in vivo antibacterial potential of SOTE [33,34].

#### 2.7.4. Immunohistochemistry

We carried out immunostaining using tumor necrosis factor-alpha (TNF-α) polyclonal antibody (Invitrogen, PA1-40281). Then, we examined TNF-α immunostaining using a light microscope, considering staining with TNF-α as a bad indication. The staining strength was classified into: absence of immunoreactive cells, (0) or negative; presence of 1–10% immunoreactive cells, (1) or weak; presence of 11–50% immunoreactive cells, (2) or moderate; and presence of more than 50% immunoreactive cells, (3) or strong [32,35].

#### 2.7.5. Measuring the Inflammatory Markers Using ELISA

The interleukins, IL-1β and IL-6, were assessed in the liver and spleen tissues as described by the manufacturer of the commercial ELISA kits (Abcam, Cambridge, UK). Their levels were revealed as pg/mL [35]. 

### 2.8. Statistical Analysis

The experiments were performed in triplicates and presented as mean ± standard deviation. The differences between the experimental groups were evaluated by one-way analysis of variance (ANOVA), followed by a post hoc test (Tukey). We constructed a Kaplan–Meier survival curve to calculate the survival rate of the rats. The difference was considered to be significant when *p* < 0.05. All these statistical calculations were carried out using Prism version 8 (GraphPad Software, San Diego, CA, USA).

## 3. Results

### 3.1. Structure Elucidation of the Isolated Compounds

**Figure 1:**
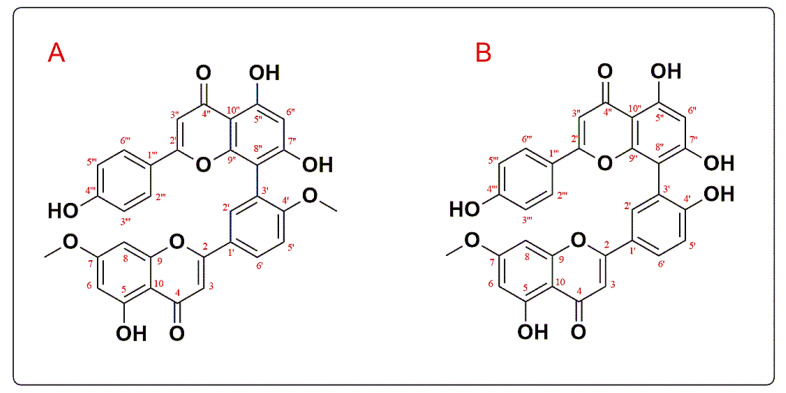
The chemical structures of GINK (**A**) and SOTE (**B**).

Compound I was established as 7,4′-*O*-dimethyl amentoflavone or ginkgetin (GINK), and compound II was 7-*O*-methyl amentoflavone or sotetsuflavone (SOTE). Their UV, ESI-MS, ^1^H, and ^13^C-NMR data were compared to those described in the literature [36,37]. GINK has UV λ_max_ (CH_3_OH) of 330 and 381, while SOTE has it of 266, 330, and 380. ESI-MS *m*/*z* 565.19 and 551.12 for the [M-H]^−^ with a molecular formula of C_32_H_22_O_10_ and C_31_H_20_O_10_, respectively. Figure 1 demonstrates the chemical structure of isolated compounds, while the ^1^H-NMR (DMSO-*d*_6_, 500 MHz) and ^13^C-NMR (DMSO-*d*_6_, 125 MHz) results are presented in Table 2. 

### 3.2. Molecular Docking Studies

**Figure 2:**
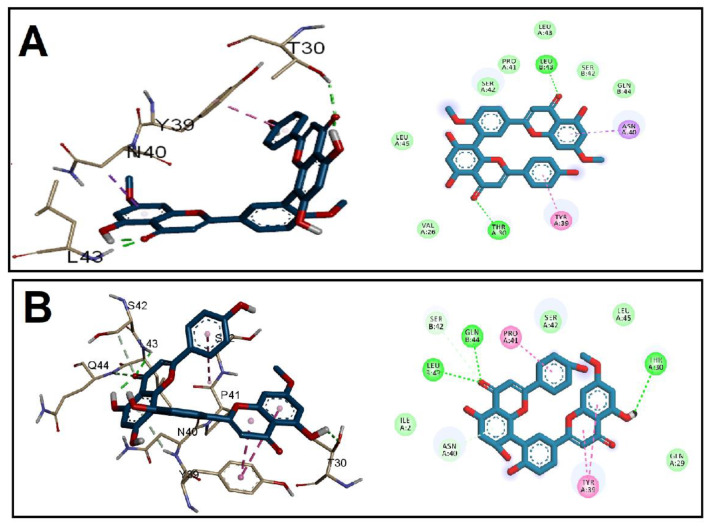
Docking of (**A**) GINK and (**B**) SOTE into the *E. faecalis* Cytolysin Regulator CylR2 receptor (Code: 1UTX).

The binding affinity and binding mechanisms for the tested GINK and SOTE on five proteins associated with *E. faecalis* virulence factors, namely, CylR2, ACE19, PrgB, ESP, and EFGE, were investigated using a molecular docking approach. The tested ligands showed high-affinity binding to the five target proteins, as indicated by the docking score (Table 1). The binding of the two drugs was nearly similar, including both hydrophobic interactions and hydrogen bonding. Docking of GINK and SOTE into the CylR2 receptor revealed a high affinity near the site for DNA binding (Figure 2). The chromone rings were engaged in H-bonding with Thr30, Leu43, and Gln44. In addition, both ligands were involved in hydrophobic interaction with Tyr39.

**Figure 3:**
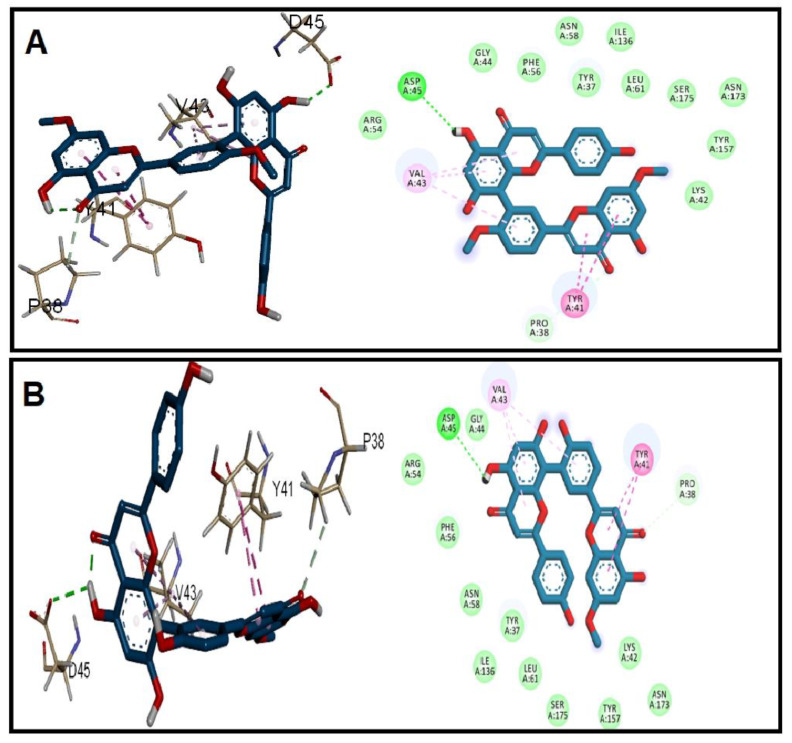
Docking of (**A**) GINK and (**B**) SOTE into the *E. faecalis* collagen-binding subdomain ACE19 (Code: 2OKM).

Regarding ACE19, the two ligands showed binding affinity to the N-terminal at the collagen peptide binding site [12]. Both ligands were bound by H-bonding with Asp45 and hydrophobic interaction through chromone ring with Tyr41 and Val43 (Figure 3). Furthermore, the docking into the PrgB, EFGE, and ESP demonstrated a slightly higher affinity of SOTE over the GINK.

**Figure 4:**
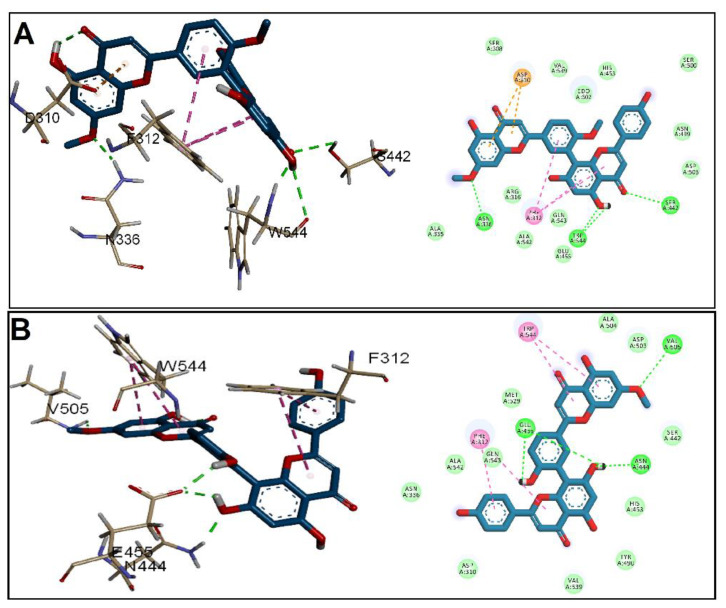
Docking of (**A**) GINK and (**B**) SOTE into the adhesin domain of PrgB from *E. faecalis* (Code: 6GED).

**Figure 5:**
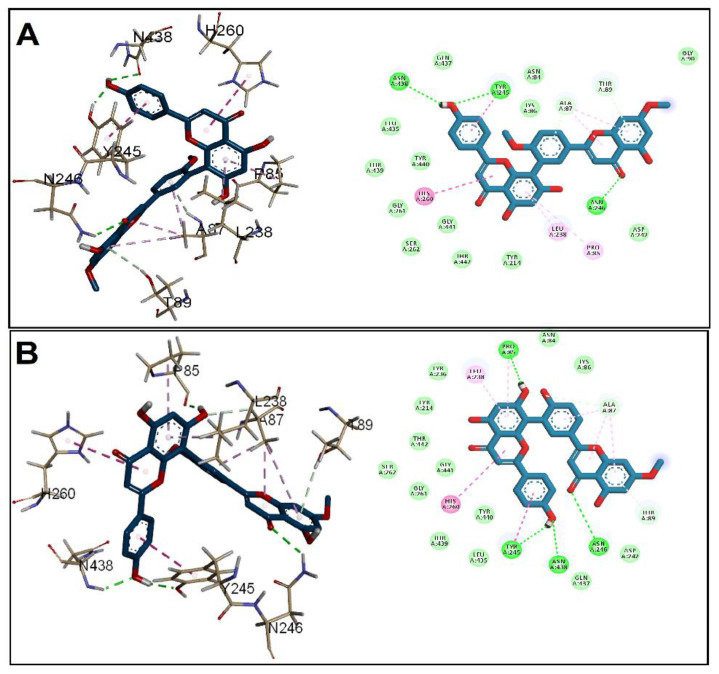
Docking of (**A**) GINK and (**B**) SOTE into the enterococcal surface protein ESP (code: 6ORI).

**Figure 6:**
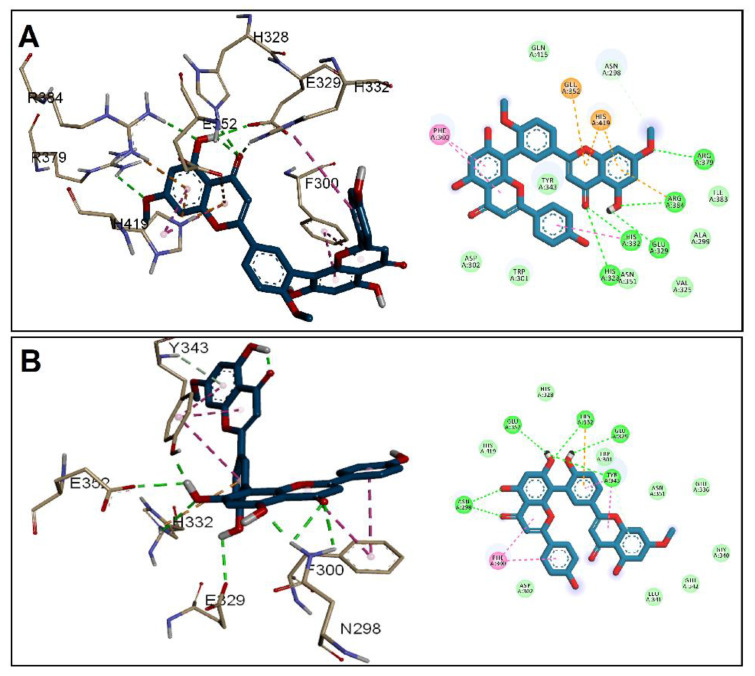
Docking of (**A**) GINK and (**B**) SOTE into the model for *E. faecalis* gelatinase enzyme EFGE (A0A7H0FPW4).

In the case of PrgB (Figure 4), GINK was at the H-bond distance with three amino acids, Asn336, Ser442, and Trp544, while Asn444, Glu455, and Val505 formed H-bonds with SOTE. Both ligands were implicated in a hydrophobic interaction with Phe312. In addition, Trp544 interacted only with SOTE, while GINK showed anion interaction with Asp310. Both ligands satisfied the interaction with His260, the proton transfer catalyst in the gelatinase EFGE, alongside other interactions presented in Figure 5. Lastly, when docking into ESP, a nearly similar interaction was found for GINK and SOTE (Figure 6), with the latter showing a higher affinity to the ESP receptor.

After all, both GINK and SOTE showed a good affinity for the five proteins enrolled in the virulence of *E. faecalis*, with SOTE being the highest, suggesting the possible mechanisms for the antivirulence activity of both ligands.

### 3.3. In Vitro Antibacterial Activity

The susceptibility of the isolates to antibiotics was explored by the Kirby–Bauer method, and their resistance profile is revealed in Table S2. The agar well diffusion method was used to evaluate the antibacterial activity of GINK and SOTE against the tested *E. faecalis* clinical isolates. Interestingly, GINK and SOTE resulted in the production of inhibition zones around the tested isolates. This finding indicates that these compounds have antibacterial activity. In addition, the determination of MIC values of SOTE and GINK by the broth microdilution method revealed that SOTE exhibited a higher antibacterial activity than GINK. This is because the MIC values of SOTE ranged from 32 to 128 µg/mL, while in the case of GINK, they ranged from 256 to 1024 µg/mL, as revealed in Table S3. 

#### 3.3.1. Membrane Depolarization

**Figure 7:**
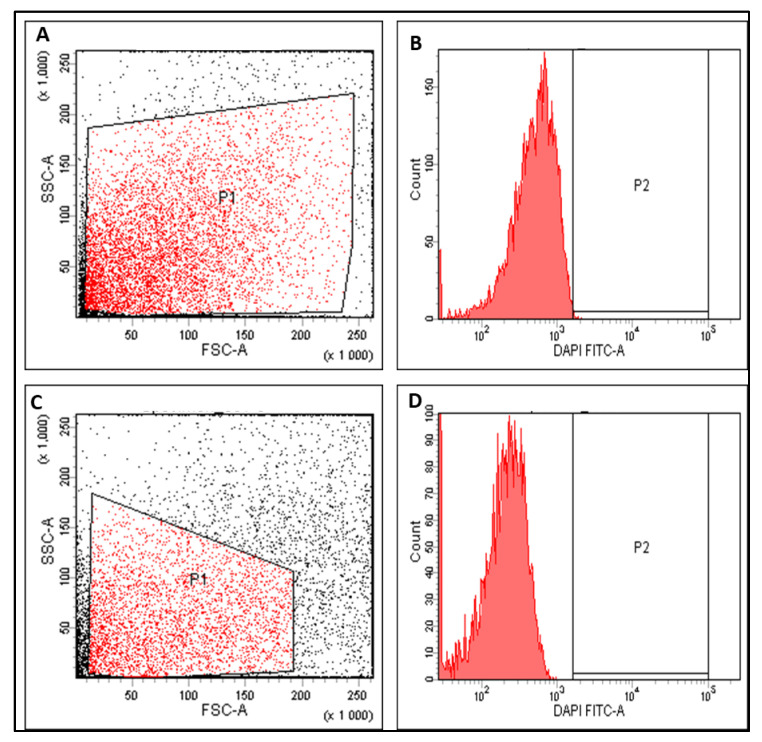
Flow cytometry charts showed a nonsignificant change in the membrane depolarization of a demonstrative *E. faecalis* isolate. Where (**A**,**B**) are before treatment and (**C**,**D**) are after treatment with SOTE.

The influence of SOTE (at the 0.5 MIC value) on the membrane depolarization of *E. faecalis* isolates was assessed using flow cytometry. The fluorescent stain (bis-(1,3-dibutylbarbituric acid) trimethine oxonol), or DiBAC4(3), was utilized to stain the isolates, as it has the ability to enter the depolarized bacterial cells where it binds to their intracellular proteins, leading to a rise in the fluorescence. SOTE did not exhibit any significant variations (*p* > 0.05) in the membrane depolarization of *E. faecalis* isolates, and a demonstrative example is revealed in Figure 7. 

#### 3.3.2. Bacterial Morphology

**Figure 8:**
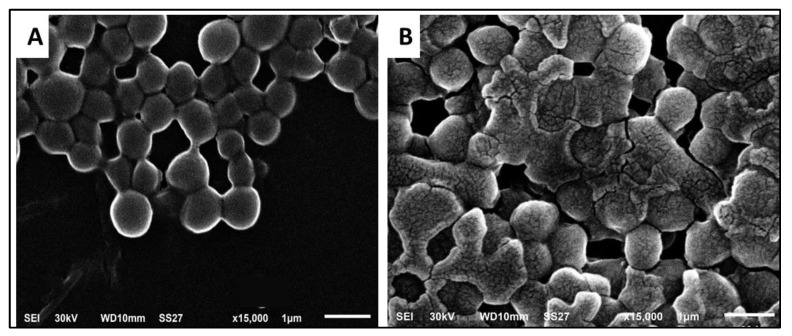
Scanning electron micrographs of a demonstrative *E. faecalis* isolate. Where (**A**) is before treatment with SOTE showing all the cells are most probably normal, with smooth surface and the cell wall of the bacterial cells is regular (×15,000); (**B**) is after treatment with SOTE, showing distortion and rupture of cell wall of some cells (×15,000).

We utilized SEM to elucidate the ultrastructural and morphological alterations in *E. faecalis* isolates that SOTE might induce. As illustrated in Figure 8, SOTE manifested distortions and deformations when compared to the cells before treatment. 

#### 3.3.3. Anti-Biofilm Activity

**Figure 9:**
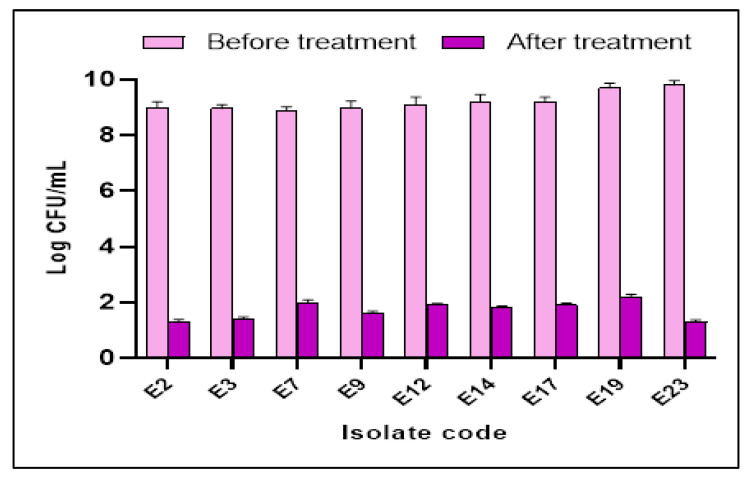
Bar chart showing *E. faecalis* isolates exhibited a significant reduction (*p* < 0.05) in the number of CFU/mL after treatment with SOTE.

The influence of SOTE (at the 0.5 MIC value) on the biofilm formation by the tested *E. faecalis* isolates was elucidated using a crystal violet assay. Interestingly, we noticed that SOTE decreased the percentage of *E. faecalis* isolates strongly and moderately forming biofilm from 60.87% to 21.74%, as revealed in Table 3. In addition, SOTE significantly reduced (*p* < 0.05) the number of CFU/mL in 39.13% of the isolates, as shown in Figure 9.

#### 3.3.4. qRT-PCR

**Figure 10:**
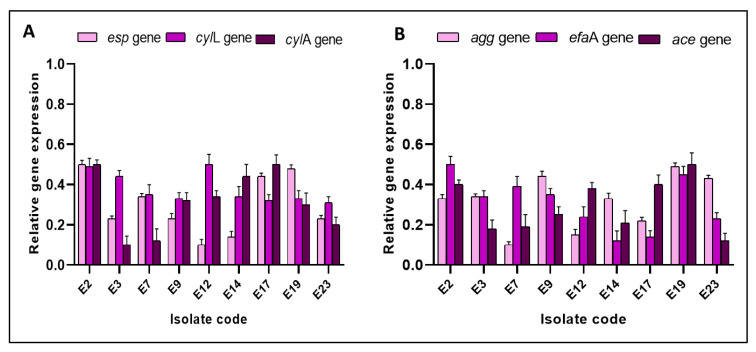
Bar charts showing *E. faecalis* isolates exhibited downregulation of the relative gene expression of (**A**) *esp*, *cyl*L, and *cyl*A, and (**B**) *agg*, *efa*A, and *ace* genes after treatment with SOTE.

Herein, we utilized qRT-PCR to elucidate the effect of SOTE on the biofilm-forming ability of the tested isolates at the molecular level. The studied biofilm-related genes in *E. faecalis* isolates were *esp*, *cyl*L, *cyl*A, *agg*, *efa*A, and *ace*. The gene *esp* was reported to enhance biofilm formation by *E. faecalis* [38]. The genes *agg*, *efa*A, and *ace* contribute to the different steps of biofilm formation [39]. Interestingly, we observed a downregulation of the expression of the studied genes in 39.13% of *E. faecalis* isolates after treatment with SOTE (0.5 MIC value), as shown in Figure 10. 

### 3.4. In Vivo Antibacterial Activity

#### 3.4.1. Bacterial Burden and Survival Curve

**Figure 11:**
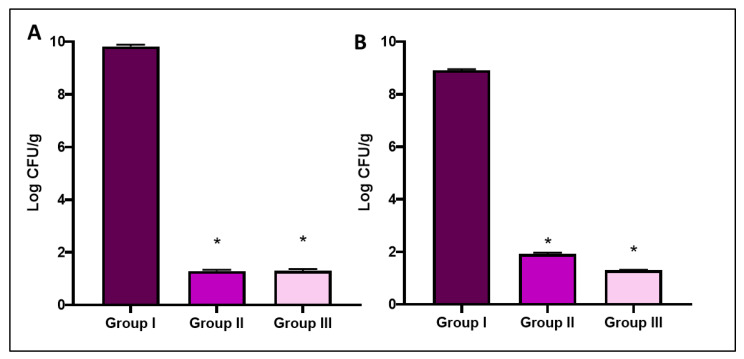
Bar charts show a significant decrease (*p* < 0.05) in the number of CFU/g in (**A**) liver and (**B**) spleen after treatment with SOTE. The symbol (*) indicates a significant change (*p* < 0.05) relative to group I.

The number of CFU/g in liver and spleen tissues was counted in all experimental groups. As revealed in Figure 11, the bacterial burden reduced significantly (*p* < 0.05) in SOTE-treated group (group III) relative to group I. 

**Figure 12:**
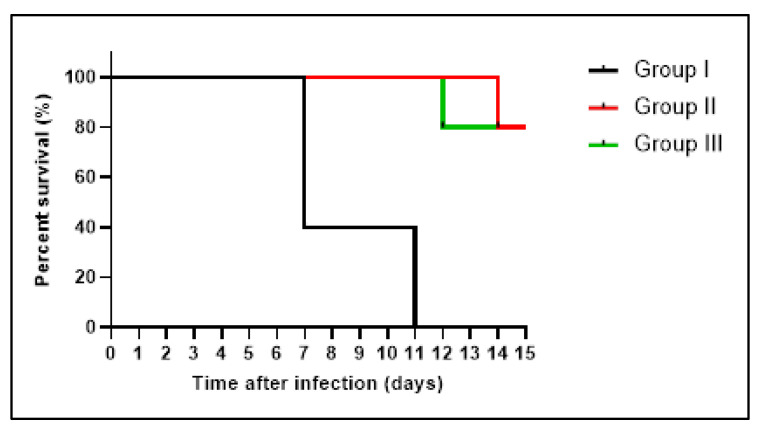
Kaplan–Meier survival curve of the experimental groups.

Furthermore, we constructed the survival curve, as revealed in Figure 12. Three rats in group I died after one week, and the remaining rats died after 11 days. Groups II and III had one rat die after two weeks and after 12 days, respectively, and the remaining rats were alive until the 20th day. 

##### Liver Function Tests

SOTE resulted in a substantial reduction (*p* < 0.05) in ALT, AST, and bilirubin levels. On the other hand, it resulted in a remarkable increase (*p* < 0.05) in total proteins and albumin levels, as shown in Table 4.

#### 3.4.2. Histological Studies

**Figure 13:**
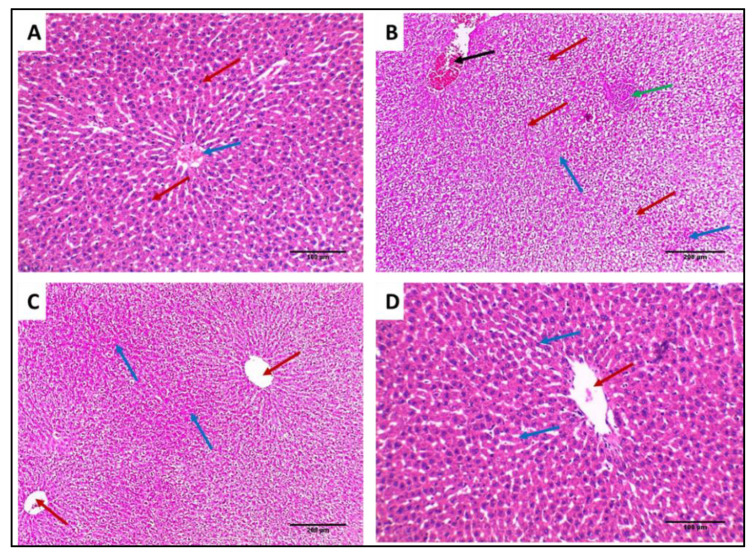
H&E-stained sections: (**A**) Normal liver showing average-sized central vein (blue arrow) surrounded by cords of hepatocytes separated by blood sinusoids (red arrows) (×200). (**B**) Liver of group I showing focal chronic inflammation (green arrow) surrounded by degenerated hepatocytes (red arrows) and spotty necrosis (blue arrows) with the congested central vein (black arrow) (×200). (**C**) Liver of group II showing average-sized central veins (red arrow) surrounded by cords of hepatocytes having focal necrosis (blue arrows) (×200). (**D**) Liver of group III showing average-sized central vein (red arrow) surrounded by cords of hepatocytes (blue arrows) (×200).

**Figure 14:**
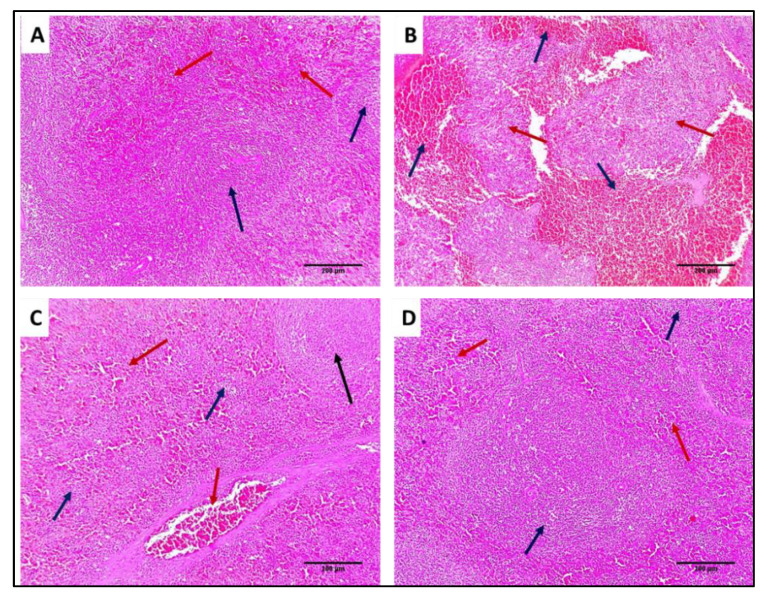
H&E-stained sections: (**A**) Normal spleen showing normal-sized white pulp (lymphoid follicles) (blue arrows) with average-sized red pulp (blood sinusoids) (red arrows) (×100). (**B**) Spleen of group I showing marked congestion exhibiting dilated congested red pulp and areas of hemorrhage (blue arrows) with destructed white pulp (red arrows) (×100). (**C**) Spleen of group II showing mild congestion in the red pulp (blue arrows) with one preserved size of lymphoid follicle (black arrow) and other some atrophic lymphoid follicles (blue arrow) (×100). (**D**) Spleen of group III showing preserved white pulp (lymphoid follicles) (blue arrows) with normal red pulp (red arrows) (×100).

**Figure 15:**
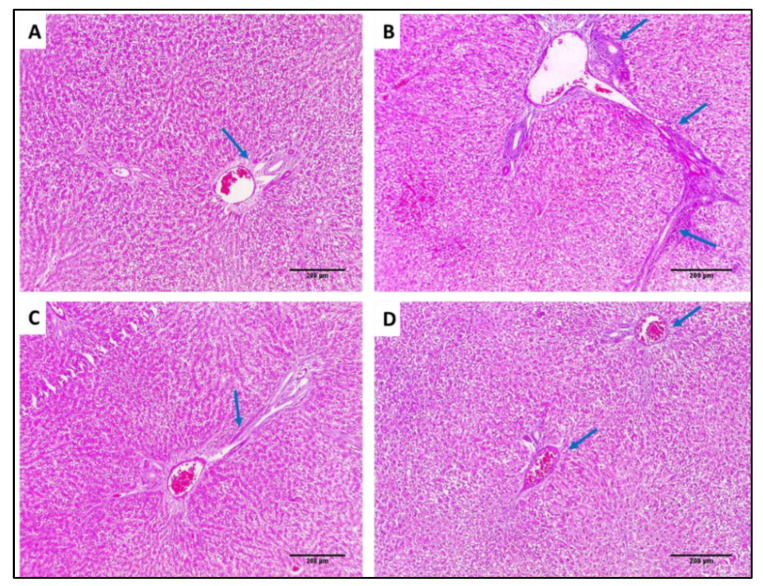
Masson’s trichrome-stained sections: (**A**) Normal liver showing portal tract with a slight fibrous wall (blue arrow), and there is no fibrosis (×100). (**B**) Liver of group I showing the fibrous expansion of portal areas with a marked portal to portal bridging (blue arrows) (×100). (**C**) Liver of group II showing some fibrous expansion of portal areas (blue arrow) (×100). (**D**) Liver of group III showing few fibrous expansion of portal areas (blue arrow) (×100).

**Figure 16:**
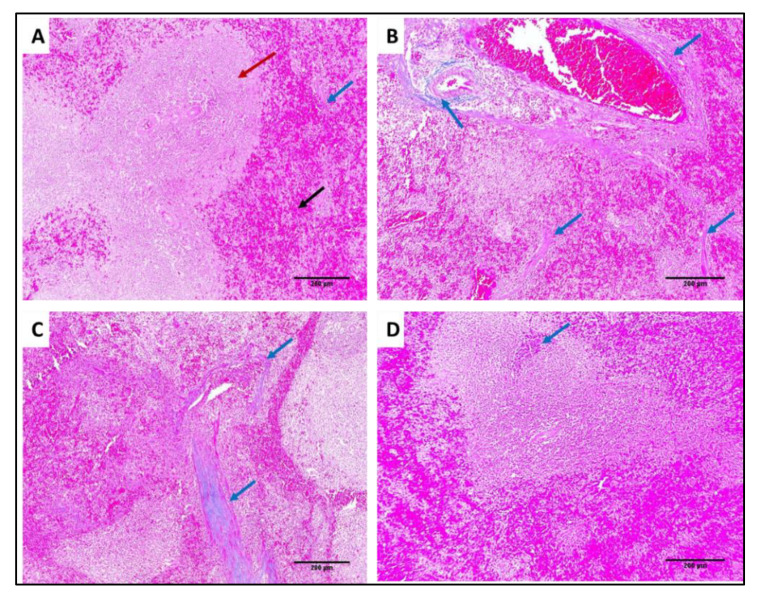
Masson’s trichrome-stained sections of: (**A**) Normal spleen showing splenic parenchyma consisting of white (red arrow) and red pulps (black arrow). The stroma is represented with slight amounts of blue-stained collagen fibers (blue arrow) (×100). (**B**) Spleen of group I showing marked congestion with marked blue-stained collagen fibers (blue arrows) (×100). (**C**) Spleen of group II showing no congestion with moderate, blue-stained collagen fibers (blue arrows) (×100). (**D**) Spleen of group III showing marked reduction of collagen fibers; only focal amount could be detected (blue arrows) (×100).

Liver and spleen tissues of the different groups were stained using H&E to assess the impact of SOTE on the tissues of rats infected with *E. faecalis,* as revealed in Figure 13 and Figure 14. In addition, collagen staining of the liver and kidney tissues from the different groups was carried out using Masson’s trichrome stain, as revealed in Figure 15 and Figure 16.

In addition, collagen staining of the liver and kidney tissues from the different groups was carried out using Masson’s trichrome stain, as revealed in Figure 15 and Figure 16.

#### 3.4.3. Immunohistochemistry

**Figure 17:**
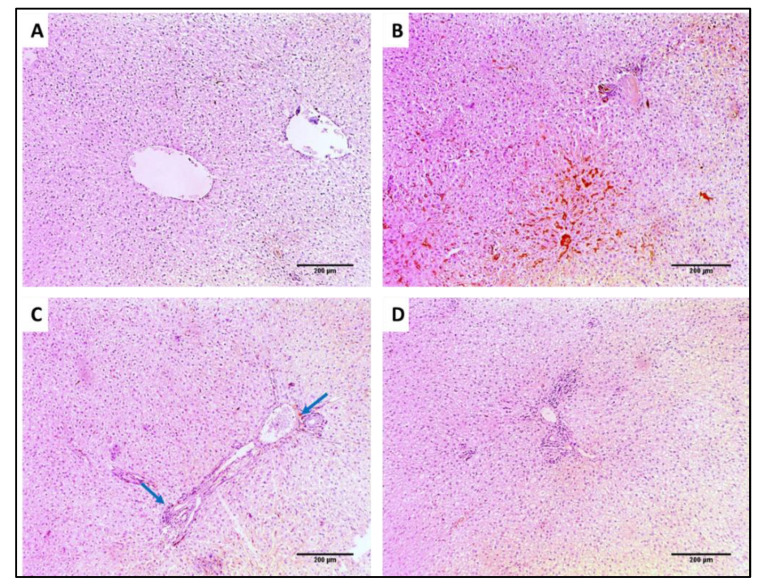
TNF-α immunostained sections of: (**A**) Normal liver showing negative TNF-α staining, score 0 (×100). (**B**) Liver of group I showing strong TNF-α staining, score 3 (×100). (**C**) Liver of group II showing weak TNF-α staining, score 1 (blue arrows) (×100). (**D**) Liver of group III showing negative TNF-α staining, score 0 (×100).

**Figure 18:**
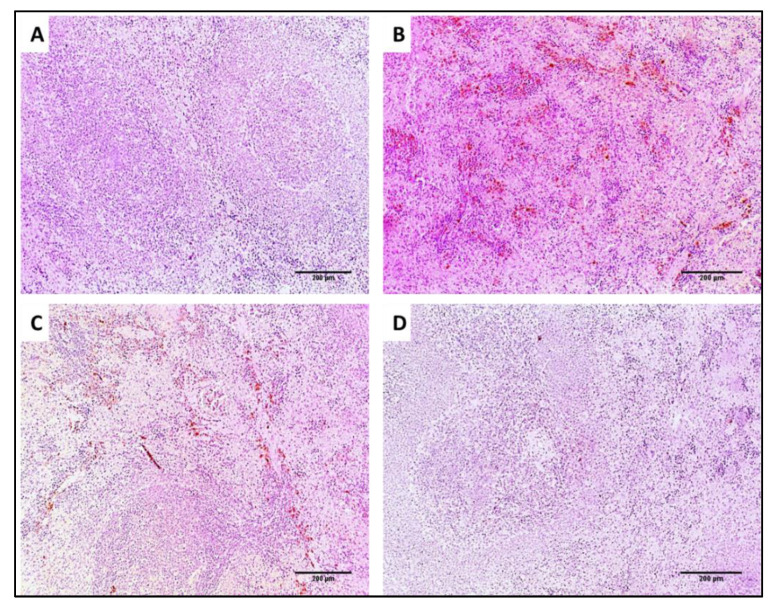
TNF-α immunostained sections of: (**A**) Normal spleen showing negative TNF-α staining, score 0 (×100). (**B**) Spleen of group I showing strong TNF-α staining, score 3 (×100). (**C**) Spleen of group II showing weak TNF-α staining, score 1 (×100). (**D**) Spleen of group III showing negative TNF-α staining, score 0 (×100).

TNF-α immunostaining of the liver and spleen tissues was performed for the different groups, as revealed in Figure 17 and Figure 18.

#### 3.4.4. Inflammation Markers

We quantified the experimental groups’ inflammation markers, interleukins (IL-1β and IL-6), in the liver and spleen tissues using enzyme-linked immunosorbent assay (ELISA). We found that the SOTE resulted in a significant decrease in their levels, as shown in Table 5.

## 4. Discussion

Public health has a major threat globally due to the disseminating antimicrobial resistance among bacterial pathogens. ESKAPE pathogens (*E. faecium*, *Staphylococcus aureus*, *Klebsiella* spp., *Acinetobacter baumannii*, *Pseudomonas aeruginosa*, and *Enterobacter* spp.) are the most common bacteria that acquire antimicrobial resistance. This issue has led to decreasing the available therapeutic options [40]. Regarding *E. faecalis,* another additional problem is its multiple virulence factors. Biofilm is a major virulence factor of *E. faecalis* that contributes to its resistance to the currently available antibiotics. Therefore, there is a high demand to find out novel treatments for infections by this pathogen [41]. 

The therapeutic activity of the naturally derived products from plants is being elucidated worldwide. This is due to the fact that plants are rich in active metabolites that can be utilized in several pharmaceutical products [41]. Thus, medicinal plants are used globally to treat various illnesses either as extracts or as isolated bioactive compounds [25]. 

For the first time, two compounds were isolated from *C. medium* R. Br dichloromethane fraction. Their biflavonoid nature was suggested by UV and ESI-MS *m*/*z*. The ^1^H NMR spectrum exhibited an AA’BB’ system by the signal at δ 7.56 (2H, d, *J* = 7.5) for H-2″′, 6″′, and δ 6.81 (2H, d, *J* = 7.5) for H-3″′, 5″′ in compound I and 7.57 (2H, d, *J* = 9), 6.70 (2H, d, *J* = 9) ascribed to H-2′’’, 6″′ and H-3″′, 5″′, respectively, in compound II. 

Also, an ABX system was displayed by signals at δ 8.06 (d, *J* = 2.5 Hz) for H-2′, 8.18 (dd, *J* = 2.5, 8.5 Hz) for H-6′, and 7.33 (d, J = 8.5 Hz) for H-5′ in compound I and 8.10 (1H, d, *J* = 2.5), 8.00 (1H, dd, *J* = 2.5, 9), and 6.98 (1H, d, *J* = 9) for H-2′, 6′, and 5′, respectively, in compound II. Regarding SO, the ^1^H-NMR spectrum revealed an amentoflavone pattern with two aromatic methoxy signals at δ 3.77 and 3.81, suggesting di-methoxy derivatives for compound I and one methoxy at **δ** 3.79 for compound II.

The ^13^C-NMR spectra found that C-8″ and C-3′ were implicated in inter-flavonoid linkage because of the downfield shift of C-8″ by 10 ppm and of C-3′ by 6 ppm, respectively. It also confirmed the biflavonoid nature by showing 30 skeleton carbons alongside methoxy signals. The ^13^C-NMR spectrum showed an upfield shift in C-5′, a downfield shift in C-1′ considering 4′-*O*-methylation, and a downfield shift in C-8 relative to amentoflavone [42]. All data are consistent with 7,4′-*O*-dimethyl amentoflavone or ginkgetin (GINK) for compound I and 7-*O*-methyl amentoflavone or sotetsuflavone (SOTE) for compound II.

The molecular docking studies of GINK and SOTE on five proteins related to *E. faecalis* virulence factors, namely, CylR2, ACE19, PrgB, ESP, and EFGE, as possible targets for the tested compound’s mechanism of action, were considered. The high docking scores (Table 1) for both tested ligands into the five target proteins indicated good affinity to the targets of interest. The binding patterns demonstrated by GINK and SOTE were nearly the same, including both hydrophobic interactions and hydrogen bonding. In general, SOTE showed a higher affinity than the GINK compound. 

We investigated the antibacterial activity of SOTE and GINK using the Kirby–Bauer method. Then, we determined the MIC values of the two compounds using the broth microdilution method against *E. faecalis*. Interestingly, we found that SOTE had lower MIC values than GINK. This finding indicates that a lower concentration of SOTE is required to exhibit antimicrobial activity against bacterial isolates than of GINK [43]. Therefore, we performed in vitro and in vivo experiments on SOTE. 

Flavonoids are polyphenolic compounds, and they are widely found in different natural plants. They are biologically active and exhibit different pharmacological activities [44]. SOTE was reported to have anticancer activity [45] via inhibition of the proliferation of the human lung cancer cells (A549). It was also reported to induce autophagy in nonsmall-cell lung cancer via blocking the PI3K/Akt/mTOR signaling pathway [44]. Regarding the activity of SOTE, according to our knowledge, this is the first study to elucidate its antimicrobial activity. Various researchers reported GINK to exhibit biological activities such as anticancer [46], anti-inflammatory, antibacterial, antifungal, antiviral, antiparasitic [37,47], antiatherosclerosis [47], and antioxidant activities [48].

We elucidated the effect of SOTE (at the 0.5 MIC value) on the membrane depolarization of *E. faecalis* isolates as the cell membrane of bacteria is a major target for multiple antibiotics. Several antimicrobial compounds exert their activity by dissipating the membrane potential either by producing ion-conducting membrane pores and enhancing the membrane ion-permeability or by acting as an ion carrier [49]. SOTE did not exhibit significant variations (*p* > 0.05) in the membrane depolarization. 

Furthermore, we investigated the impact of SOTE on bacterial morphology using SEM. This resulted in the manifestation of distortions and deformations in the treated cells. Finally, it is reported that many antibacterial compounds affect the morphology and ultrastructure of the bacterial cells by causing deformations in the cell wall and leakage of the intracellular components [50].

Though the main focus in developing novel antimicrobials is on their ability to inhibit bacterial growth, another approach focuses on the antivirulence activity. Interestingly, some antibiotics can affect bacterial virulence in addition to their growth inhibition ability. For instance, it was reported that linezolid could diminish the virulence factors’ production from *S. aureus* [50,51]. Thus, we also explored the anti-biofilm activity of SOTE by crystal violet assay and qRT-PCR. 

Antimicrobial compounds are usually more effective when they also have anti-biofilm activity. They will be able to penetrate the formed biofilms, exert their antibacterial activity, and diminish biofilm formation [51]. SOTE decreased the percentage of *E. faecalis* isolates strongly and moderately, forming biofilm from 60.87% to 21.74%. Moreover, it caused a downregulation in the studied biofilm-related genes (*esp*, *cyl*L, *cyl*A, *agg*, *efa*A, and *ace*) in 39.13% of *E. faecalis* isolates. Previous studies have reported the anti-biofilm activity of flavonoids. Matilla-Cuenca et al. [48] reported that the flavonoids such as quercetin, myricetin, and scutellarein inhibit a biofilm-associated protein that mediates biofilm formation of *Staphylococcus aureus* and other staphylococcal species. Additionally, Raorane et al. [52] reported that three flavonoids, fisetin, phloretin, and curcumin, exhibited a dose-dependent inhibition of the biofilm formation by *Acintobacter baumannii*.

In addition, we inspected the in vivo antimicrobial activity of SOTE using a systemic infection model in rats. SOTE resulted in a remarkable decrease in the bacterial burden in the liver and spleen. It also improved the liver function tests and raised the survival rates among the infected rats. We noticed a decrease in congestion, bleeding, and inflammation using H&E and Masson’s trichrome staining of the liver and spleen. 

In an attempt to explain these effects, we quantified IL-1β and IL-6 in the liver and spleen tissues using ELISA. In addition, we examined the TNF-α immunostaining of the liver and spleen. SOTE was found to reduce all these inflammation markers significantly. In sepsis induced by bacterial pathogens, the inflammatory cytokines such as IL-1β, IL-6, and TNF-α function as major mediators for shock and death induction. This means that the profuse production of these cytokines is the leading cause of the fatal consequences of sepsis [53].

## 5. Conclusions

Phytochemical investigation of DCM fraction of *Cycas media* R. Br leaves resulted in the isolation of GINK and SOTE for the first time. The molecular docking studies suggested five proteins (CylR2, ACE19, PrgB, ESP, and EFGE) related to *E. faecalis* virulence factors as possible mechanisms illustrating the antivirulence activity of GINK and SOTE. It also predicted higher activity of SOTE compared to GINK, which was supported by the experimental findings. Remarkably, SOTE exhibited greater antimicrobial activity against bacterial isolates compared to GINK. SOTE significantly affected the morphology and ultrastructure of the treated *E. faecalis* cells by triggering deformation in the cell wall and leakage of the intracellular components. In addition to its growth inhibition ability, SOTE showed a potent antivirulence activity, revealing a notable anti-biofilm activity. Furthermore, SOTE significantly downregulated the inflammatory cytokines IL-1β, IL-6, and TNF-α in the in vivo experiment. These cytokines are major mediators for shock and sepsis, which induce death. 

As a final point, this work sheds light on *C. media* R. Br as a rich source of bioactive metabolites with potent antibacterial and antivirulence activities. This could be utilized in several pharmaceutical products as alternative treatment strategies for infections caused by *E. feacalis* and to reduce the fatal consequences of sepsis.

## Figures and Tables

**Table 1:** The grid box parameter (center and size) and docking binding affinity (Kcal/mL) of GINK and SOTE for five E. faecalis virulence factor proteins.

Receptor	Grid Box (x, y, z)		Affinity (kcal/mol)	
	Center	Size	GINK	SOTE
CylR2	23.43, −4.93, 12.79	20.0, 20.0, 15.0	−7.4	−7.5
ACE19	3.4, −12.7, 83.3	25.9, 24.9, 22.8	−7.4	−7.6
PrgB	36.8, 23.3, 20.6	52.8, 66.1, 61.1	−8.9	−9.6
EFGE	−0.6, −1.8, 3.1	33.7, 34.1, 32.3	−9.5	−9.9
ESP	0.9, −4.0, 0.1	41.6, 45.3, 47.3	−10.7	−11.1

**Table 2:** ^1^H-NMR and ^13^C-NMR (DMSO-*d_6_*, 500 and 125 MHz) for the isolated compounds.

Compound I (GINK)			Compound II (SOTE)	
δ-H		δ-C	δ-H	δ-C
2		163.7		164.2
3	6.92 (1H, s)	103.1	6.80 (1H, s)	102.8
4		181.9		181.9
5		161.5		161.1
6	6.19 (1H, d, *J* = 1.5 Hz)	98.1	6.34 (1H, d, *J* = 2.5 Hz)	98.1
7		165.1		163.4
8	6.49 (1H, d, *J* = 1.5)	92.7	6.77 (1H, d, *J* = 2.5)	92.6
9		157.3		157.5
10		104.7		102.8
1′		122.3		120.9
2′	8.06 (1H, d, *J* = 2.5)	128.2	8.10 (1H, d, *J* = 2.5)	127.6
3′		121. 7		121.5
4′		160.6		160.5
5′	7.33 (1H, d, *J* = 8.5)	111.7	6.98 (1H, d, *J* = 9)	117.0
6′	8.18 (1H, dd, *J* = 2.5, 8.5)	130.9	8.00 (1H, dd, *J* = 2.5, 9)	131.5
2″		163.7		164.4
3″	6.78 (1H, s)	102.6	6.88 (1H, s)	102.5
4″		182.1		182.1
5″		160.4		160.9
6″	6.38 (1H, s)	98.2	6.33 (1H, s)	99.4
7″		161.6		161.9
8″		103.7		104.7
9″		154.5		154.5
10″		103.6		103.1
1″′		120.9		120.9
2″′	7.56 (2H, d, *J* = 7.5)	128.0	7.57 (2H, d, *J* = 9)	128.1
3″′	6.81 (2H, d, *J* = 7.5)	115.8	6.70 (2H, d, *J* = 9)	115.7
4″′		161.1		160.2
5″′	6.81 (2H, d, *J* = 7.5)	115.8	7.57 (2H, d, *J* = 9)	115.7
6″′	7.56 (2H, d, *J* = 7.5)	128.0	6.70 (2H, d, *J* = 9)	128.1
OCH_3_	3.77, 3.81	56.1, 55.9	3.79	56.1

**Table 3:** Influence of SOTE on biofilm-forming ability of the tested *E. faecalis* isolates.

Biofilm Forming Ability	Number of the Isolates	
	Before Treatment with SOTE	After Treatment with SOTE
Non-biofilm forming	5	10
Weak	4	8
Moderate	8	3
Strong	6	2

**Table 4:** Assessment of the liver function in the tested groups.

**Groups**	ALT (U/L)	AST (U/L)	Bilirubin (mg/dL)	Total Proteins (g/dL)	Albumin (g/dL)
Group I	92.0 ± 2.3	135.1 ± 4.3	0.56 ± 0.04	1.8 ± 0.1	2.1 ± 0.3
Group II	46.2 ± 3.2 *	70.0 ± 2.5 *	0.15 ± 0.001 *	7.0 ± 0.4 *	4.45 ± 0.3 *
Group III	46.0 ± 2.2 *	71.2 ±1.2 *	0.16 ± 0.002 *	6.9 ± 0.3 *	4.44 ± 0.2 *

Data are expressed as mean ± standard deviation. The symbol (*) represents a significant difference (*p* < 0.05) in comparison with group I.

**Table 5:** Levels of IL-1β and IL-6 in the different experimental groups.

Tissues	Liver		Spleen	
Inflammation Markers	IL-1β Level (pg/mL)	IL-6 Level (pg/mL)	IL-1β Level (pg/mL)	IL-6 Level (pg/mL)
Group I	73.3 ± 1.2	200.5 ± 5.2	71.1 ± 2.0	195.4 ±4.3
Group II	23.2 ± 2.3 *	100.2 ± 3.4 *	24.1 ± 1.4 *	99.1 ± 3.2 *
Group III	22.6 ± 2.3 *	110.1 ± 4.1 *	23.9 ± 1.5 *	98.2 ± 4.3 *

Data are expressed as mean ± standard deviation. The symbol (*) represents a significant difference (*p* < 0.05) in comparison with group I.

## Data Availability

Data are contained within the article and Supplementary Materials.

## Supplementary Materials

The following supporting information can be downloaded at: https://www.mdpi.com/article/10.3390/antibiotics11080993/s1, Table S1. Sequences of the utilized primers in the current study. Table S2. MIC values of GINK and SOTE against *E. faecalis* isolates. Table S3. MIC values of GINK and SOTE against E. faecalis isolates. Scheme S1. Extraction and fractionation steps of *Cycas media* R. Br leaves. Scheme S2. Column chromatography of *Cycas media* DCM fraction.
