# Supplementary material for: Anti-Biofilm and Antibacterial Activities of Cycas media R. Br Secondary Metabolites: In Silico, In Vitro, and In Vivo Approaches
Source: Antibiotics (Basel). 2022 Jul 24;11(8):993. doi: 10.3390/antibiotics11080993 (PMC9394325; doi:10.3390/antibiotics11080993)
Supplement: Supplementary file 1 [file antibiotics-11-00993-s001.zip › antibiotics-1821021-supplementary.pdf]

Table S1. Sequences of the utilized primers in the current study.

Genes	Oligonucleotide sequences (5′-3′)
esp	F: CGGTCATACCGACGACCAAA<br>R: TGTCACATCGCCATCGACTT
*cyl*L	F: GATGGAGGGTAAGAATTATGG<br>R: GCTTCACCTCACTAAGTTTTATAG
*cyl*A	F: ACTCGGGGATTGATAGGC<br>R: GCTGCTAAAGCTGCGCTT
Agg	F: CACGTAATTCTTGCCCACCA<br>R: AAACGGCAAGACAAGTAAATA
*efa*A	F: GACAGACCCTCACGAATA<br>R: AGTTCATCATGCTGTAGTA
Ace	F: CAAGCATTATTGGCAGCGTT<br>R: TCTATCACATTCGGTTGCG
16s rRNA (housekeeping gene)	F: CCGAGTGCTTGCACTCAATTGG<br>R: CTCTTATGCCATGCGGCATAAAC

Table S2. Resistance profile of the tested isolates.

Isolate code	Resistance profile*	Isolate code	Resistance profile*
E1	CN-E-TE	E13	AMP-C-TE
E2	E-AMP-C	E14	CN-E-AMP-AMC-C
E3	E-AMP-AMC	E15	CN-AMP-C
E4	AMP-TE	E16	AMP-C
E5	AMP-AMC	E17	E-AMP-TE
E6	AMP-C-CIP	E18	CN-E-AMP-AMC-C
E7	E-AMP-TE	E19	AMP-TE
E8	TE	E20	CN-E-AMP
E9	CN-E-AMP-AMC-CIP-VA	E21	TE
E10	E-AMP-TE	E22	CN-E-AMP-AMC-C-CIP-VA
E11	AMP	E23	E-AMP-C-TE
E12	E-TE		

* Gentamicin (CN), erythromycin (E), ampicillin (AMP), amoxicillin/clavulanic acid (AMC), chloramphenicol (C), ciprofloxacin (CIP), tetracycline (TE), vancomycin (VA), and linezolid (LZD).

Table S3. MIC values of GINK and SOTE against *E. faecalis* isolates.

Isolate code	MIC of GINK (µg/mL)	MIC of SOTE (µg/mL)
E1	256	32
E2	256	32
E3	512	32
E4	512	128
E5	512	64
E6	256	64
E7	1024	32
E8	256	64
E9	1024	128
E10	1024	128
E11	1024	32
E12	256	32
E13	1024	32
E14	256	32
E15	512	64
E16	256	128
E17	1024	64
E18	256	128
E19	1024	32
E20	1024	64
E21	512	64
E22	512	128
E23	512	128

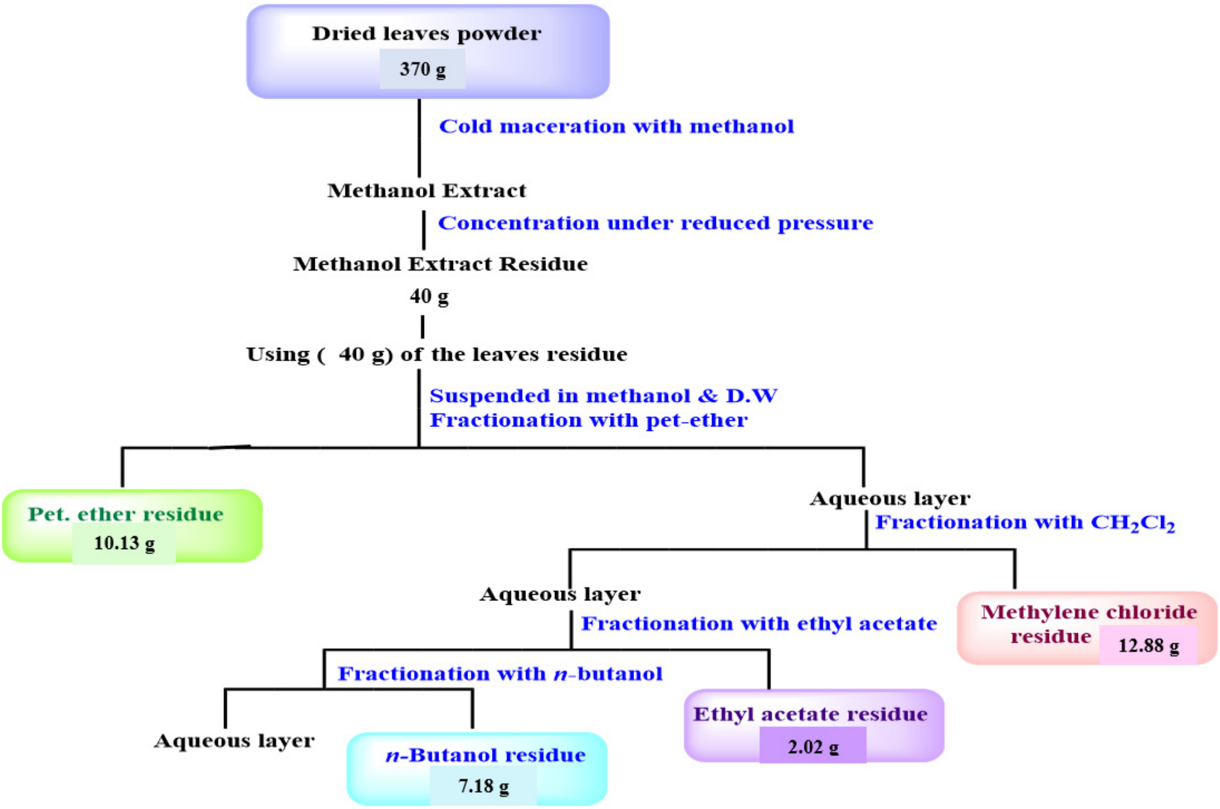

Scheme (S1): Extraction and fractionation steps of *Cycas media* R. Br leaves

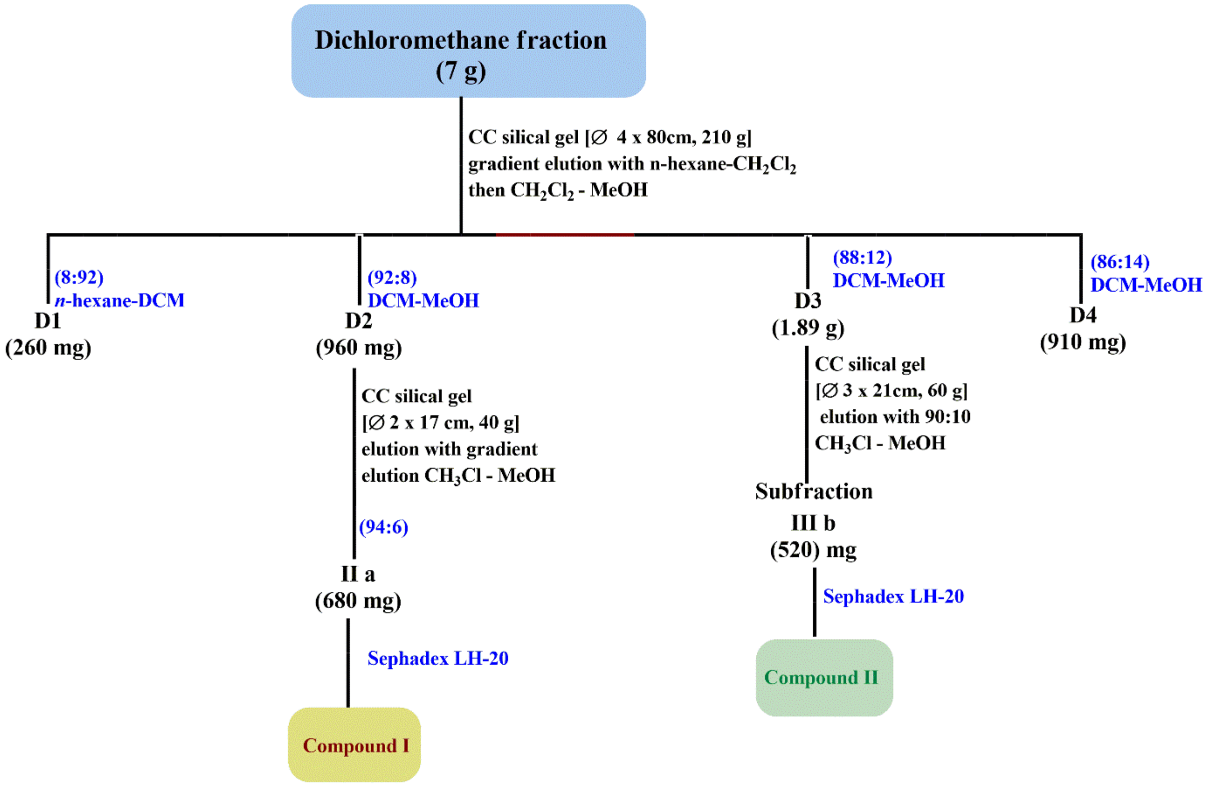

Scheme (S2): Column chromatography of *Cycas media* DCM fraction
